# Endoscopic resection of a huge tumor located at an anatomically challenging position using an innovative extra snare traction method

**DOI:** 10.1055/a-2261-7794

**Published:** 2024-03-01

**Authors:** Xin-Guang Cao, Saif Ullah, Shan-shan Zhu, Nan Dai, Chang-Qing Guo

**Affiliations:** 1 Department of Gastroenterology, The First Affiliated Hospital of Zhengzhou University, Zhengzhou, China; 2 Department of Gastroenterology, The First Affiliated Hospital of Zhengzhou University, Zhengzhou, China

**Fig. 1:**
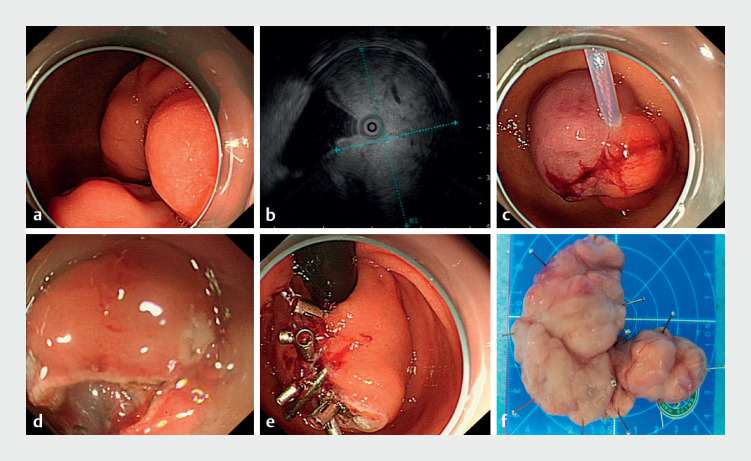
Method for endoscopic removal of a huge duodenal tumor. **a** Upper gastrointestinal endoscopy showed a huge irregular mass occupying the entire duodenal lumen. **b** Endoscopic ultrasound showed intact submucosal and muscular layers. **c** A snare was used to pull the tumor into the gastric cavity via the pyloric opening. **d** The lesion was resected. **e** The wound was closed using endoclips. **f** The resected specimen.

A 56-year-old man was admitted to our hospital with a 20-day history of intermittent abdominal pain. Enhanced computed tomography of the abdomen revealed a huge submucosal mass in the duodenal bulb. Upper gastrointestinal endoscopy showed a huge irregular mass occupying the entire duodenal lumen, making it impossible to visualize the complete extent under endoscopy, indicating inevitable traumatic open surgery ( Fig. 1 **a** ). However, endoscopic ultrasound showed intact submucosal and muscular layers, suggesting the possibility of endoscopic resection ( Fig. 1 **b** ). After obtaining patient consent and explaining the standard of care alternatives, we decided to perform endoscopic resection of the lesion.

Owing to the enormous size of the tumor and unclear operative field, it was impossible to operate within the duodenal lumen. Innovatively, we employed a snare to pull the tumor into the gastric cavity via the pyloric opening, as follows: 1) introduced the snare through the biopsy channel; 2) looped and secured the snare around the head of the tumor; 3) dragged the tumor into the gastric cavity ( Fig. 1 **c** ); and finally 4) used scissors to cut the snare near the handle to maintain tension on the tumor, and then withdrew the endoscope leaving the snare in place. ( Video 1 ). The lesion was then resected using endoscopic submucosal dissection ( Fig. 1 **d** ). The specimen (9.5×8.5 cm) was removed carefully with assistance from the snare ( Fig. 1 **e** ).

Endoscopic resection of a huge tumor located at an anatomically challenging position using an innovative extra snare traction method.Video 1

The resected specimen was sent for histopathology examination, which confirmed the diagnosis of Brunner’s adenoma ( Fig. 1 **f** ). At 3 months’ follow-up, gastroscopy confirmed that the wound had completely healed, and no lesions remained.

We report this innovative method in which a huge duodenal tumor was pulled into the gastric cavity, making the otherwise difficult-to-perform procedure simple and effective, thereby avoiding the need for traumatic open or laparoscopic surgery. We believe that this method can also be applied to resect huge tumors in anatomically challenging locations within the gastrointestinal tract. This case can be useful to colleagues who meet similar situations and may avoid a referral to open traumatic surgery.

Endoscopy_UCTN_Code_TTT_1AO_2AG

